# Teacher knows best? The social influence of teachers and peers in high school

**DOI:** 10.1111/jora.70063

**Published:** 2025-08-17

**Authors:** Ana da Silva Pinho, Scarlett Slagter, Andrea Gradassi, Lucas Molleman, Barbara R. Braams, Wouter van den Bos

**Affiliations:** ^1^ Ontwikkelingspsychologie Universiteit van Amsterdam Amsterdam the Netherlands; ^2^ Afdeling Klinische Neuro‐en Ontwikkelingspsychologie Vrije Universiteit Amsterdam Amsterdam the Netherlands; ^3^ Max‐Planck‐Institut Fur Bildungsforschung Berlin Germany

**Keywords:** adolescence, intentions, norms, peer influence, personal norms, teacher influence

## Abstract

Social norms are crucial to human development and social functioning. They play an important role in the formation of personal norms and intentions concerning risk‐taking and prosocial behaviors. During adolescence, the school becomes a prominent environment where individuals gain a deeper understanding of broader social norms, helping them navigate the complexities of adult society. Here, adolescents may learn social norms from two key sources: peers and teachers. While heightened sensitivity to peers is well established, less is known about the influence of teachers in shaping adolescent personal norms and intentions. We performed a pre‐registered two‐wave experiment (*N* = 270; *M*_age_ = 13.7 years old) to investigate how normative information from peers and teachers directly and indirectly shapes intentions for risk‐taking and prosocial behaviors. Results from our moderated mediation analysis indicate that normative information influences intentions for risk‐taking and prosociality both directly and indirectly by shaping personal norms. Furthermore, while the source does not moderate the direct effect, it does moderate the indirect pathway by influencing personal norms. In particular, adolescents tend to conform more strongly to peer norms and exhibit anti‐conformity toward teacher norms in the context of risk‐taking. Overall, our findings highlight the often overlooked importance of teachers in co‐shaping social norms during these formative years.

## INTRODUCTION

Social norms are typical patterns and rules within a group that play a significant role in shaping individuals' personal norms and intentions, guiding behavior crucial to human development and social functioning (Bicchieri, 2005; Cialdini et al., 1991; Cialdini & Goldstein, 2004). Early in development, children learn to adjust their behavior by learning norms from parents or guardians (Bandura, 1971), who serve as the primary source of transmitting knowledge and skills to their child (vertical social learning; Schniter et al., 2023). As they transition into adolescence, social influence shifts toward same‐aged peers (horizontal social learning; Albert et al., 2014; Crone & Dahl, 2012; Fuhrmann et al., 2015; Malonda et al., 2019; Pinho et al., 2021; Slagter, Gradassi, et al., 2023; Slagter, van Duijvenvoorde, & van den Bos, 2023) and other adults, such as teachers (oblique social learning; Demps et al., 2012; Farine et al., 2015; Gurven et al., 2020; Hewlett, 2021; Lew‐Levy et al., 2023). Despite robust evidence on the role of social influence during adolescence (e.g., Blakemore & Mills, 2014; Crone & Dahl, 2012), experimental studies investigating how adolescents adjust their intentions in response to real normative information from peers and teachers remain scarce. This study addresses this gap by employing an experimental design to examine how peer and teacher norms shape adolescents' intentions across two socially relevant behavioral domains: risk‐taking and prosociality.

The literature suggests that adolescents' heightened social sensitivity also extends to social norms, which often guide behavior toward socially desirable outcomes, particularly by reducing risk‐taking and increasing prosociality (Pinho et al., 2021). Risk‐taking involves behaviors with potential negative outcomes, such as reckless driving or alcohol consumption, often driven by reward sensitivity (Steinberg, 2008), and peaks when in the presence of peers (Chein et al., 2011). Prosocial behavior, characterized by voluntary actions benefiting others, such as helping or sharing (Eisenberg et al., 2006) also plays an important role during adolescence, influenced by moral reasoning (Kohlberg, 1984; Turiel, 2006), social responsibility (Fuligni, 2019), and the need to matter and care (Dahl & Killen, 2024).

As adolescents spend a lot of time at school, it becomes a prominent environment for their identity and social development (Eccles & Roeser, 2011; Roeser et al., 2000; Verhoeven et al., 2019). This suggests that both peers and teachers play crucial roles in the development of social norms for risk‐taking and prosociality during adolescence. While the impact of peers on adolescents' own norms and intentions is well‐established, less is known about the influence of teachers. The aim of our study is to experimentally examine how normative information shapes intentions for risk‐taking and prosocial behavior, both directly and indirectly through personal norms. Additionally, we examine how these pathways are moderated by the source providing the norms: peers and teachers.

### Direct effect of normative information on intention

To understand the relative impact of peers and teachers on adolescents' own norms and intentions for real‐world behaviors, we focus on two pathways proposed by the literature through which social norms shape behavior via the development of these intentions. Intentions—a person's likelihood to take an action (Ajzen, 1991, 2020)—are strong predictors of both risk‐taking and prosocial behaviors, such as alcohol use (Cutrín et al., 2020) and blood donation (Bednall et al., 2013), respectively. In the first pathway, social norms can *directly* impact intentions. That is, individuals change their intentions by simply complying with the social norm without altering their personal norm. This compliance, which is externally reinforced, may be driven by the desire to obtain social approval or the fear of being punished if individuals violate the social norm (Cialdini & Goldstein, 2004). For instance, adolescents conform to peer norms about aggressive behavior to avoid exclusion from the group as these norms are socially accepted within the classroom (Laninga‐Wijnen et al., 2017; Rambaran et al., 2013). Hence, examining the direct influence of normative information from peers and teachers helps us understand whether social compliance contributes to adolescents' intentions to engage in risk‐taking and prosocial behavior.

### Personal norms as a mediator between normative information and intention

In the second pathway, social norms can *indirectly* impact intentions through a process of norm updating, where individuals first alter their personal norms, which are attitudes about the appropriateness of behavior in given situations (Cialdini et al., 1991). In this case, individuals would follow the social norm because they are internally motivated to align their personal norms with those of the group (Davis et al., 2018; Gavrilets & Richerson, 2017; Gintis, 2003). Internal signals, such as guilt, could function as an intrinsic motivation for norm compliance (Battigalli & Dufwenberg, 2007) but also internal rewards, such as pride in achieving social values and ideals (Chudek & Henrich, 2011). For instance, peer norms can impact individuals' intention for waste prevention (Balundė et al., 2020) or for recycling (White et al., 2009) via personal norms, and this may be helpful for the school and the worldwide environment. This type of compliance can have a prolonged impact on intentions, particularly if individuals do it even without being observed by others.

### Moderation effect of the source: the influence of peers and teachers

The extent to which adolescents adjust their intentions for risk‐taking or prosocial behavior may depend on the source of social norms, in particular, whether it originates from peers or teachers. Learning from peers is particularly important in understanding what is typically (dis)approved of in the school context, including key behaviors such as risk‐taking and prosociality (Lapinski & Rimal, 2005; Pinho et al., 2021; Telzer et al., 2018). Conforming to peer norms often rewards adolescents with social status and acceptance; if they deviate from these norms, they can face punishments, such as exclusion (Asch & Asch, 1956; Cialdini & Goldstein, 2004; Dijkstra et al., 2008; Rambaran et al., 2013). Hence, the norms set in the classroom can affect adolescents' socio‐emotional functioning and behavioral development. For instance, peer norms that encourage bullying have been associated with increased bullying in the classroom (Sentse et al., 2015), while peer norms endorsing prosociality lead adolescents to be more approving of prosocial actions, such as helping others (Pinho et al., 2021).

At the same time, teachers may serve as role models for adolescents, exerting influence on their learning and social development (Hendrickx et al., 2016; Roshandel & Hudley, 2018; Vollet et al., 2017). For instance, teachers can foster cohesive relationships between students (Farmer et al., 2019; Hamm & Hoffman, 2016), and the quality of their relationships with adolescents can promote motivation and academic engagement (Kilday & Ryan, 2019; Quin, 2017; Ruzek et al., 2016). Teachers may also support adolescents to change or reinforce norms, particularly when these norms are challenging or reflect complex dynamics in the classrooms (Engels et al., 2020; Veenstra & Lodder, 2022), such as enhancing inclusive behavior (Engels et al., 2020). While most of the evidence indicates that teachers are influential in the prosocial domain, their impact on the risk‐taking domain is less understood (Yoon & Bauman, 2014). However, given their significant influence on adolescent development, teachers may be key targets for interventions aimed at lessening risk‐taking and strengthening prosociality.

### The current study

**FIGURE 1:**
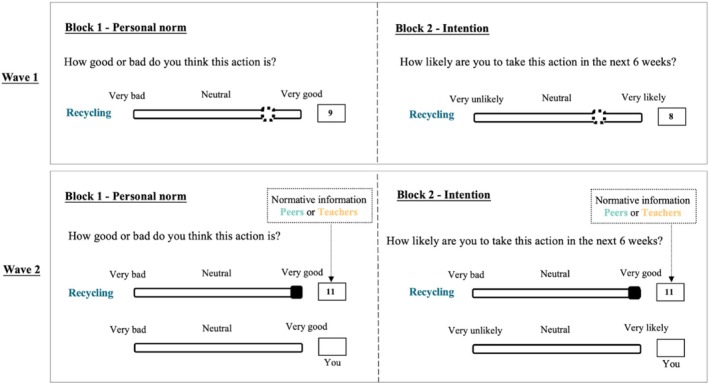
Experimental design. Top panel—wave 1: We measured participants' personal norms (PN_1_; ranging from 1 = very bad to 11 = very good) and intentions (IB_1_; ranging from 1 = very unlikely to 11 = very likely) of a set of risk‐taking and prosocial behaviors (e.g., recycling) in wave 1. Bottom panel—wave 2: Participants observed normative information (NI = 11) from either peers or teachers regarding a risk‐taking or a prosocial behavior (in this case recycling) and rated their personal norms (PN_2_, represented as ‘*You*’) and intentions (IB_2_, represented as ‘*You*’) again. The order of the behavioral items within each block as well as the order of the blocks was randomized between individuals in both waves.

Our pre‐registered study investigates how peers and teachers shape adolescents' intentions for risk‐taking and prosocial behaviors, using a controlled two‐wave experimental design (*N* = 270; Figure 1). Most studies on adolescent social influence rely on observational data (e.g., Rambaran et al., 2013; Van Ryzin & Dishion, 2017), which provide valuable insights but may introduce potential confounds, such as selection bias (Manski, 1993), making it difficult to isolate the magnitude or the direction of the effects. By experimentally controlling the availability of normative information, we directly compare the influence of peers and teachers on adolescents' intentions while minimizing these confounds inherent in observational studies.

In the first wave, adolescents rated their personal norms and intentions, and teachers indicated their (dis)approval of adolescents' behavior. In the second wave, adolescents observed norms from either peers or teachers and rated their personal norms and intentions again. The observed social norms signaled more disapproving of risk‐taking and more approving of prosociality than adolescents' initial norms. This experimental manipulation allowed us to examine the influence of peer and teacher normative information on personal norm and intention formation. Throughout this manuscript, we use the terms peer norm and teacher norm interchangeably to refer to a group's social norm or normative information, representing the aggregated personal norms of individuals within that specific group.

We expected (1) normative information to directly shape intention change, (2) updates in personal norms to be a significant mediator of the relationship between normative information and intentions, (3) source to be a significant moderator of both direct and indirect paths. Particularly, we expected peers to be more influential than teachers, given that belonging to the peer group is highly important during adolescence (LaFontana & Cillessen, 2010; Tomova et al., 2021). However, the opposite outcome could also be possible; previous research comparing peers of the same age and adults in the risk domain yielded mixed results (e.g., Knoll et al., 2017), or found no differences between the sources in the prosocial domain (Foulkes et al., 2018).

Lastly, we conducted exploratory analyses to examine whether individual differences moderate the effects of normative information. First, we tested whether age moderates both the direct and indirect pathways, based on prior evidence that adolescents' susceptibility to social influence varies developmentally. For instance, young adolescents are more susceptible to their peers than adults in a risk perception task (ages: 12–14; Knoll et al., 2017), and resistance to peer influence increases from early to late adolescence (ages: 14–18; Steinberg & Monahan, 2007). We expected the effect of normative information from peers to be more pronounced with younger adolescents, and teachers to be more influential with older adolescents. Second, we explored the moderating role of adolescents' subjective closeness to the group from which the norm stemmed (Aron et al., 1992) to capture potential individual differences in perceived social affiliation with the influencing source.

## METHODS

### Participants

Based on a power analysis, we would have needed to recruit a sample of 200 participants to ensure statistical power at the level of 80% to detect the hypothesized moderated indirect effect of normative information on intention (see Statistical analyses, and Table S1 for more details). Given the two‐wave design of the study and the possibility of having dropouts between the waves, as well as the availability of the participating high schools, we recruited an initial sample of 324 adolescents. From this initial sample, 14 participants had missing responses due to technical issues, being distracted in wave 1, or incorrectly inserted their identifier not enabling us to match the corresponding data in wave 2; 40 were absent from their class in wave 2. This led to a final dataset that consisted of 1924 observations (consisting of 930 experimental trials reflecting risk‐taking behaviors and 994 reflecting prosocial behaviors) from 270 participants (*M*_age_ = 13.7, SD = 1.51; 47% identified as female, 46% male, 6% did not report, and 1% other) from 18 classrooms divided into 10 1st year of high school (*n* = 170) and eight 3rd year of high school (*n* = 100). Including these high school grades in our sample aligned with key developmental transitions. First‐year students (early adolescence) undergo social adjustments and a lot of exploration when entering high school, while third‐year students (mid‐adolescence) tend to explore a broader range of behaviors (Ciranka & van den Bos, 2021), making the study of social norm development and intention formation particularly relevant. However, it is important to highlight that logistical constraints, such as school availability and the demands of a two‐wave design, limited recruitment, particularly of older adolescents.

### General procedure

Prior to the study, we obtained informed consent from all participants. For minors, we additionally obtained informed consent from their parents or legal guardians. The study took place at Dutch high schools in a classroom setting. Data were collected in 2 waves with a two‐ to three‐week interval. We kept the regular classroom setup to minimize disruption and keep it as naturalistic as possible, except for the study equipment on the students' desks. Before each testing session, the researchers placed tablets and screen dividers on desks so students had privacy to perform the experiment. Every testing session began with oral instructions by the experimenters, followed by possible questions regarding the study. After that, students performed the experiment using the tablets. Each session lasted approximately 30–45 min, and schools received a monetary compensation of €5 per participant for each wave. Additionally, to enhance performance and voluntary participation, one participant per class was randomly selected to win a €10 online shop voucher. Participating teachers performed the task online on their own device. Communication with teachers occurred through the school. They received two emails via the school: (1) an invitation to participate in the study with an information letter about the experiment; (2) an email with the link for the experiment in which they could only proceed to the study after providing informed consent. All procedures were approved by the Ethics Review Board Faculty of Social and Behavioral Sciences of the University of Amsterdam.

### Experiment—wave 1

The current approach builds on previous research that experimentally studied personal norms during adolescence (e.g., Pinho et al., 2021) and extends it to investigate changes in intentions. The task consisted of two blocks (see Figure 1). In one block, participants rated their (dis)approval of 36 actions (example of risk‐taking item: smoking; example of prosocial item: helping others; see Table S2 for a full list of items) on an 11‐point scale from 1 (very bad) to 11 (very good). This measured their personal norms (PN). In the other block, they indicated their likelihood of performing these same 36 actions in the next 6 weeks on an 11‐point scale from 1 (very unlikely) to 11 (very likely). If an action was not applicable, adolescents could press a button and the action would disappear. This block measured their intention of behavior (IB). The order of the items within each block as well as the order of the blocks was randomized between individuals. Teachers were asked to indicate their (dis)approval of adolescents' behavior by rating the same 36 actions on an 11‐point scale from 1 (very bad) to 11 (very good). For pre‐registered study design and hypotheses, see https://osf.io/h3ny9/?view_only=c35b74a29125413e949cede4906f45bb.

### Experiment—wave 2

The task again comprised two blocks. In each block, participants rated six risk‐taking and six prosocial behaviors from both the group of peers and the group of teachers. Within each block, we balanced the number of trials (i.e., behavioral items) in each domain (risk and prosociality). Across trials, we systematically varied the normative information to be either more disapproving of risk‐taking behaviors or more approving of prosocial behaviors than participants' personal norms in wave 1. Previous research has shown that socially desirable peer norms are highly influential (Pinho et al., 2021). We therefore focused on peer and teachers' norms signaling socially desirable directions. That is, stronger disapproval of risk‐taking and stronger approval of prosociality than participants' initial personal norms.

Each block consisted of 12 trials, eight experimental trials and four filler trials which were interspersed between the experimental trials. Filler trials consisted of normative information which was the same or close to participants' original ratings. These filler trials were included as a control mechanism to minimize potential desirable demand characteristics in their responses and enhance the believability of the study by preventing participants from detecting the experimental manipulations. We randomized the block order between participants, and within each block we randomized the order of experimental trials (i.e., extreme social norm (peers vs. teachers) relative to participants' initial personal norm) and the filler trials. The information shown to participants reflected the actual norm from a group of peers and a group of teachers who have completed the task in wave 1 (albeit we selectively displayed the source's ratings to create the experimental conditions; see below). The group consisted of the mode rating for an item and was communicated as “the most chosen rating in a group of classmates” and “the most chosen rating in a group of teachers”.

To determine wave 2 trials, we used two criteria to ensure that participants could have room to change their personal norms and intentions in socially desirable directions (that is, increasing disapproval of risk‐taking and increasing approval of prosocial behavior). First, we targeted items in which participants' initial response indicated moderate disapproval of risk‐taking actions (e.g., PN_1_≈[3–5]) and moderate approval of prosocial actions (e.g., PN_1_≈[7–9]). To determine the group norm shown to participants, we selected a group between 5 and 7 participants within each class as well as between 5 and 7 teachers out of a group of 15 teachers such that their most chosen rating was 2 points higher (for prosocial items) or 2 points lower (for risk‐taking items) than a participant's original personal norm. This approach held the distance between PN_1_ and normative information constant. Second, for each domain, we avoided trials in which participants had extreme initial ratings of intention (i.e., IB_1_ = 1 and IB_1_ = 11). This was done to ensure that participants could update their intentions, if they wanted, in response to the normative information observed. This setup ensured experimental control using real information.

### Measures

Normative information is the independent variable and represents the distance between the group mode rating for a particular item and participants' initial personal norm (*N* = NI − PN_1_). Participants' personal norm change (ΔPN) is the mediating variable and it represents the difference between participants' personal norms in wave 2 (PN_2_) and in wave 1 (PN_1_). Negative ΔPN values mean personal norm weakening, and positive ΔPN means personal norm strengthening; particularly, we expected personal norm weakening in the risk‐taking domain and personal norm strengthening in the prosocial domain. Participants' intention change (ΔIB) is the dependent variable and it represents the difference between participants' intention in wave 2 (IB_2_) and in wave 1 (IB_1_). Positive and negative ΔIB values follow the same criteria as described for ΔPN. The source of information is the moderator and consists of peers and teachers.

#### Subjective closeness

We measured perceived closeness to the group of peers and the group of teachers from which the norm was derived. Participants filled in a short visual questionnaire based on the Inclusion of the other in the Self scale (IOS), ranging from 1 (very distant) to 7 (very close; Aron et al., 1992). This scale has been shown to have high test–retest reliability and convergent validity (Aron et al., 1992; Gächter et al., 2015). Although we did not pre‐register hypotheses regarding the impact of subjective closeness on both direct and indirect pathways, we included this measure to test for potential individual differences in perceived closeness to the source of the normative information as exploratory analysis.

### Statistical analyses

All statistical analyses were conducted in R, RStudio v. 1.3.1093 (RStudio Team, 2020). We used *pwrSEM*, v0.1.2 (Wang & Rhemtulla, 2021) to conduct a power analysis for the hypothesized moderated indirect effect of normative information on intention change. We used teachers as reference for source (0; peers = 1), set up values for the parameters of the hypothesized model (see Table S1) and ran 10,000 simulations. The values for these parameters were based on previous literature (which includes moderate direct effect and small indirect effect; Doran & Larsen, 2016; Pinho et al., 2021). Assuming a significance level of α = .05, a sample consisting of around 200 participants would ensure that statistical power is at least 80% for detecting the hypothesized moderated indirect effect.

**FIGURE 2:**
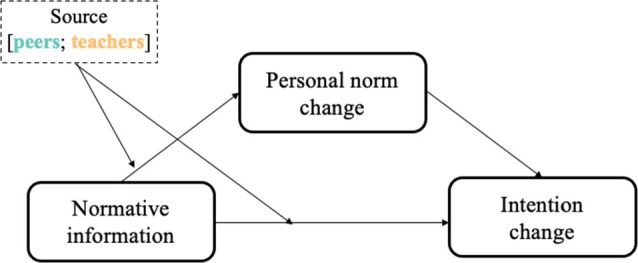
Conceptual moderated mediation model testing the impact of normative information on intention, both directly and indirectly through personal norms, with the moderation effect of the source of normative information.

Before conducting the preregistered main analysis, we performed descriptive analyses including paired sample *t*‐tests to examine mean‐level differences between wave 1 and wave 2 in personal norms and intentions for risk‐taking and prosociality, and then by source. Following our main analysis plan, we tested whether the source of normative information moderated the direct and indirect effects of normative information on intentions (see Figure 2 for conceptual model), using the *sem* function of the *lavaan* package (Rosseel, 2012).

The model was estimated incorporating a clustering variable to account for distinct school classrooms. We used the robust cluster‐adjusted standard errors (*robust. cluster*) in the *sem* function (Rosseel, 2012), which corrects standard errors for within‐cluster correlations. This adjustment accounts for the non‐independence of observations within clusters, leading to more accurate standard errors and improved robustness of parameter estimates in the presence of clustered data (Liang & Zeger, 1986). Additionally, we applied the Satorra‐Bentler scaled chi‐square test (*satorra.bentler*) in the *sem* function (Rosseel, 2012) which is a robust test that corrects the chi‐square statistic and standard errors to provide more reliable inference in the presence of non‐normal data or moderate‐to‐large samples (Satorra & Bentler, 1994, 2001). By applying this adjustment, we improve the reliability of model evaluation, making it more robust to potential violations of normality assumptions.

Lastly, to test the significance of the indirect effect, we further conducted the same analysis but included a non‐parametric bootstrap confidence interval with 1,000 simulations, selecting the *bootstrap* test and *robust* standard errors from the *sem* function instead of the robust cluster‐adjusted standard errors. In order to test whether outliers or extreme cases influenced the results, we removed 27 observations (leading to 1897 observations but keeping the sample of 270 participants) in which the moves in personal norm were lower or higher than 1.5 times the interquartile range, and reran the analysis. The obtained results were similar to those including the whole dataset. Therefore, the main results obtained are not due to extreme cases in norm updates. We thus kept all observations in the dataset for the analyses presented below.

Our theoretically informed model included all hypothesized paths, making it fully saturated (df = 0). As a result, the model perfectly reproduces the observed data, meaning conventional fit indices, such as the Root Mean Square Error of Approximation (RMSEA) and the Comparative Fit Index (CFI) are not informative because they will indicate perfect fit (CFI = 1, RMSEA = 0; Hu & Bentler, 1999; Kline, 2005; Weston & Gore, 2006). In such cases, model fit cannot be evaluated using standard goodness‐of‐fit measures because a fully saturated model does not impose any constraints on the data (Kline, 2015). Instead, model interpretation relies on theoretical justification and parameter estimates, rather than overall fit indices.

Finally, as part of our exploratory analyses, we conducted two separate moderated mediation models, one for each behavioral domain: risk‐taking and prosociality. These analyses, which were not preregistered, aimed to further investigate potential differential mechanisms in response updates to normative information across source and domain. Additionally, we explored the potential moderating influence of age and subjective closeness on both the direct and indirect effects of normative information on intention change. These exploratory analyses were conducted using the *sem* function of the *lavaan* package (Rosseel, 2012).

## RESULTS

### Descriptive analyses

Table 1 presents the descriptive statistics for personal norms and intentions for risk‐taking and prosociality across Wave 1 and Wave 2. Significant mean‐level differences were observed between the waves, particularly in personal norms. After being exposed to normative information in Wave 2, participants reported greater disapproval of risk‐taking behaviors and increased approval of prosociality. Regarding intentions, a significant difference was found only for prosocial intentions, with participants indicating a greater likelihood of engaging in prosocial behavior compared to their initial ratings. However, no significant change was observed for risk‐taking intentions.

**TABLE 1:** Descriptive statistics of personal norms and intentions for risk‐taking and prosociality between Wave 1 and Wave 2.

	Wave 1, *M* (SD)	Wave 2, *M* (SD)	df	*t*	*p*	*p* (Bonferroni)	Cohen's *d*
Risk‐taking							
Personal norms	5.55 (2.23)	4.86 (2.77)	929	−10.64	<.001	<.001	−0.35
Intention	5.42 (3.16)	5.16 (3.27)	929	−3.23	.001	.012	−0.11
Prosociality							
Personal norms	6.57 (2.21)	7.58 (2.63)	993	15.99	<.001	<.001	0.51
Intention	4.70 (2.79)	6.16 (3.05)	993	16.67	<.001	<.001	0.53

*Note*: Means (*M*) and standard deviations (SD) are reported for each wave. Negative *t*‐values indicate a decrease from Wave 1 to Wave 2. *p*‐values were adjusted using the Bonferroni correction for multiple comparisons (α = .004). Cohen's *d* represents effect sizes, with 0.2 = small, 0.5 = medium, and 0.8 = large (Cohen, 1988).

**FIGURE 3:**
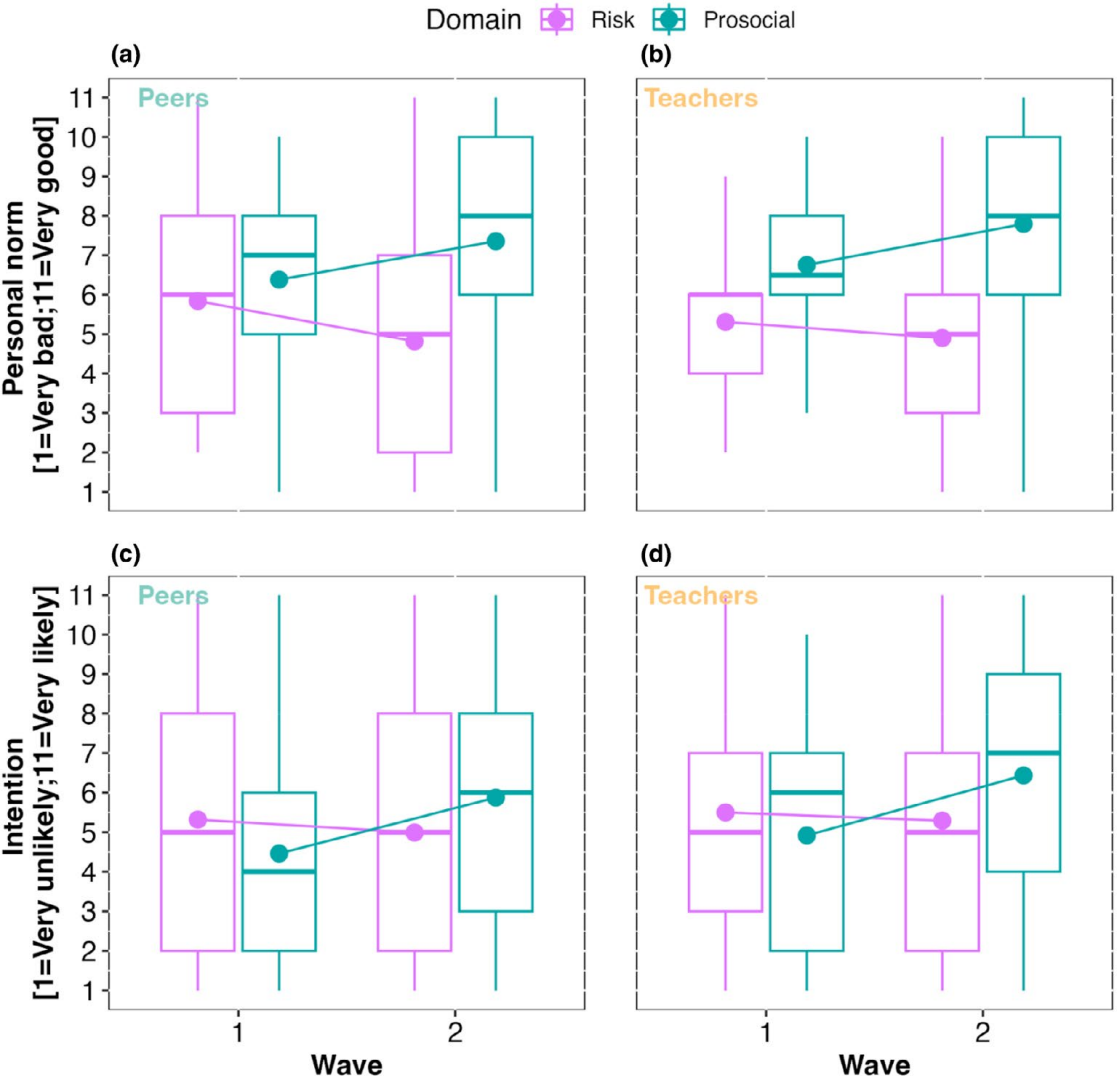
(a) Participants' personal norms of risk‐taking (purple) and prosocial (turquoise) behaviors, for Waves 1 and 2. The boxplots represent the interquartile range (IQR), the median is represented by the horizontal line and the whiskers include the smallest and largest values within 1.5 times the IQR. After observing normative information from peers in wave 2, participants became more disapproving or risk‐taking behavior and more approving of prosociality. (b) The same pattern was visible when normative information stemmed from teachers. (c) Participants' intentions across domains (risk‐taking in purple and prosocial in turquoise), for waves 1 and 2. After observing normative information from peers in wave 2, participants reported they would be less likely to engage in risky behavior and more likely to engage in prosociality than in wave 1. (d) Similar behavior was observed when normative informing came from teachers. See Figures S1a,b and S2a,b for distributions in personal norms updates and intentions updates.

Additionally, the descriptive statistics for the study variables, broken down by source (peers and teachers), reveal mean‐level differences between waves (see Table 2 and Figure 3a–d). Specifically, observing peer norms in Wave 2 influenced participants' personal norms, leading to greater disapproval of risk‐taking and increased approval of prosocial behavior. Similarly, exposure to teachers' norms in wave 2 led participants to express more disapproval of risk‐taking and more approval of prosociality. Regarding intentions, a significant shift was observed only for prosocial intentions. After exposure to normative information, participants expressed a greater likelihood of engaging in prosocial behavior, regardless of whether the source was peers or teachers.

**TABLE 2:** Descriptive statistics of personal norms and intentions for risk‐taking and prosociality between Wave 1 and Wave 2, broken down by source: Peers and teachers.

	Wave 1, *M* (SD)	Wave 2, *M* (SD)	df	*t*	*p*	*p* (Bonferroni)	Cohen's *d*
Peers							
Risk‐taking							
Personal norms	5.84 (2.59)	4.82 (3.02)	439	−6.81	<.001	<.001	−0.32
Intention	5.32 (3.41)	4.99 (3.38)	439	−2.44	.015	.18	−0.12
Prosociality							
Personal norms	6.38 (2.50)	7.36 (2.85)	467	11.06	<.001	<.001	0.51
Intention	4.46 (2.78)	5.88 (3.08)	467	10.54	<.001	<.001	0.49
Teachers							
Risk‐taking							
Personal norms	5.31 (1.87)	4.90 (2.55)	489	−8.23	<.001	<.001	−0.37
Intention	5.50 (2.95)	5.29 (3.17)	489	−2.14	.033	.40	−0.10
Prosociality							
Personal norms	6.75 (1.87)	7.79 (2.39)	525	11.52	<.001	<.001	0.50
Intention	4.92 (2.79)	6.44 (3.00)	525	12.99	<.001	<.001	0.57

*Note*: Follow the same criteria as in Table 1.

### Main analyses

#### Moderated mediation model of behavioral intentions

Table 3 shows the results of the hypothesized model examining whether intentions for risk‐taking and prosociality are influenced both directly by normative information and indirectly through personal norm change, and whether these pathways are moderated by the source of the information. The results indicate that intention change was directly impacted by normative information; however, this effect did not depend on the source. Furthermore, personal norm change was a statistically significant mediator between normative information and intention change. As a robustness check, we also performed a bias‐corrected bootstrap confidence interval procedure with 1000 simulations, which confirmed the indirect effect (95% CI = [0.05, 0.14]). However, the way this normative information is adopted to alter individuals' own norms is different depending on the source. In other words, the source is a statistically significant moderator of the impact of normative information on intention change via changing personal norms. Particularly, individuals seem to conform more to normative information from peers than teachers. Overall, normative information impacts intentions directly and indirectly. While the impact of both sources in shaping intentions is similar through the direct pathway, their influence differs via the indirect pathway (Figure S3, for response strategies in personal norm updates). This suggests that key sources in adolescents' social networks may impact them differently depending on the route of influence.

**TABLE 3:** Impact of normative information on intentions during adolescence.

	Estimate (SE)	*p*
Direct effect		
Normative information→Personal norm change (*a*)	0.25 (0.05)	<.001
Personal norm change→Intention change (*b*)	0.38 (0.03)	<.001
Normative information→Intention change (*c'*)	0.31 (0.08)	<.001
Indirect effect		
a*b	0.09 (0.02)	<.001
Total effect		
*c'* + *ab*	0.41 (0.08)	<.001
Moderator		
Source on path *c'*	–0.06 (0.05)	.202
Moderator		
Source on path *ab*	0.04 (0.01)	.002
RMSEA	0	
CFI	1	

*Note*: Estimates from the moderated mediation model fitted to changes in intention. Normative information serves as the independent variable, intention change as the dependent variable, and personal norm change as the mediating variable. Source is the moderator. The hypothesized model examines whether intentions are influenced directly by normative information (path c') and indirectly through personal norm change (mediation), as well as whether these pathways are moderated by the source. This explains 19% of the variance in personal norm change (*R* ^2^ = 0.19) and 17% of the variance in behavioral intentions change (*R* ^2^ = 0.17). The model reports unstandardized coefficients. SE represents standard error.

### Exploratory analyses

Given the moderating effect of source on personal norm change, we further examined differential underlying mechanisms by conducting a moderated mediation analysis separately for each behavioral domain: risk‐taking and prosociality. Regarding risk‐taking (Model 1, Table S3), neither the direct effect of normative information on intentions nor the indirect effect via personal norm change reached statistical significance. Furthermore, no significant moderation effect of source was observed on either pathway (see Figure S3, left panel, for response strategies in personal norm updates related to risk‐taking).

For prosociality (Model 2, Table S3), findings suggest that the effect of normative information on prosocial intentions primarily operates through changes in personal norms. Additionally, we found no evidence of differential effects based on the source; that is, teachers and peers exerted equal influence (see Figure S3, right panel, for response strategies in personal norm updates related to prosociality). However, these findings should be interpreted with caution, as splitting the data may reduce statistical power to detect the hypothesized moderated mediation effects. This is reflected in the increased standard errors observed in the separate models compared to the preregistered model.

### Individual differences

#### Moderating effect of age

Contrary to our expectations, age does not moderate the direct effect nor the indirect effect of normative information on intention change when it stems from peers (Model 1 in Table S4). When normative information is provided by teachers, age moderates only the direct influence of teachers' norms on intention change, with this effect reducing as age increases (Model 2 in Table S4).

#### Moderating effect of perceived closeness

Finally, adolescents' perceived closeness to the group of peers (*M* = 5.25, SD = 1.72, Figure S4a) does not moderate the direct and indirect effects of normative information on changes in intentions (Model 1 and 2 in Table S5). Perceived closeness to the group of teachers (*M* = 4.66, SD = 1.97; see Figure S4b) does not significantly moderate the indirect effect but does influence the direct pathway. In particular, the direct effect of normative information on intention change was stronger among individuals who felt closer to teachers.

## DISCUSSION

Our study investigated how peers and teachers shape adolescents' norms and intentions for risk‐taking and prosocial behaviors. More specifically, we examined the specific pathways in which adolescents' sensitivity to normative information from peers and teachers occurs, and present three main findings. First, intentions are directly influenced by normative information from peers and teachers alike. Second, our moderated mediation analysis showed that normative information shapes intentions indirectly, by first altering adolescents' personal norms. Third, adolescents' response strategies in personal norm updates suggest that while peers and teachers are equally influential in shaping prosociality, their influence diverges in the risk‐taking domain, with adolescents conforming more to peers and anti‐conforming to teachers. Together, these findings suggest that both sources play a crucial role in shaping adolescents' personal norms and intentions. However, their influence depends on the pathway through which they operate, whether external or internal, as well as the specific behavioral domain. In the following sections, we address each of these findings and discuss their implications.

### Direct effect of normative information on intention

In line with our expectations, normative information had a positive direct effect on changes in adolescents' intentions. This result is consistent with existing literature suggesting the strong influence of norms on intentions (e.g., Sheeran et al., 2016). From a developmental perspective, the direct use of normative information to adjust intentions may enable individuals to quickly align their behavior with social expectations (Cialdini & Goldstein, 2004). Adjusting intentions based on social approval or disapproval can be an effective strategy for obtaining social rewards, such as validation and group belonging (Telzer et al., 2021), while also avoiding sanctions, such as the risk of exclusion from the social group (Cialdini & Goldstein, 2004; Laursen & Veenstra, 2021). Thus, individuals may find it more cost‐effective to update their actions in response to social signals indicating (dis)approval of behavior rather than risk making social errors.

On one hand, given that our design promoted socially desirable behaviors, such as rejecting risk‐taking and endorsing prosociality, this can serve as an adaptive strategy for adolescents, facilitating the rapid adjustment of their actions in an appropriate way to changing social environments (Blakemore & Mills, 2014; Saragosa‐Harris et al., 2022). On the other hand, this process may not always be beneficial in the real world, as blindly conforming to normative information can also lead to maladaptive decision‐making, particularly when the conveyed norms are undesirable, conflict with personal values, long‐term goals, or accurate risk assessments (Ciranka & van den Bos, 2021; van de Bongardt et al., 2015). Beyond modifying their intentions in response to social norms, adolescents may also first engage in a process of internal reflection and norm assimilation (Van Hoorn et al., 2018), which can lead to updates in personal norms.

### Personal norms as a mediator between normative information and intention

Indeed, we observed that changes in adolescents' intentions are shaped indirectly via altering personal norms. This aligns with norm internalization theories (Cialdini & Trost, 1998; Lapinski & Rimal, 2005), which suggest that external social norms also influence behavior if they are internalized as personal norms. Although our two‐wave experimental design did not allow for a direct assessment of internalization, the observed updating of personal norms suggests that adolescents are willing to revise their personal standards based on external influences. Furthermore, prior research indicates that personal norms can act as a bridge between social influence and decision‐making (Ajzen, 1991; Rimal & Real, 2003). For instance, in the domain of prosocial behavior, studies have shown that exposure to prosocial norms encourages behavioral change when individuals internalize those norms as personally meaningful (Padilla‐Walker & Carlo, 2014; Van Hoorn et al., 2018).

Although adolescence is a period characterized by heightened sensitivity to social norms and peer approval (Blakemore & Mills, 2014; Piehler, 2011; Saragosa‐Harris et al., 2022), this heightened sensitivity develops in parallel with increasing autonomy and the maturation of self‐concept (Pfeifer & Berkman, 2018). As a result, while adolescents are highly sensitive to social influences, they simultaneously develop a greater capacity for independent evaluation and self‐reflection. This dual developmental process may account for the mediation effect observed in our study. Particularly, as adolescents refine their self‐concept, they do not merely adopt external norms but undergo cognitive deliberation; processing and evaluating social information before adopting it (Klaczynski, 2001).

### Moderation effect of the source: the influence of peers and teachers

While the direct effect of normative information was independent of its source, the way individuals adopted this information to reshape their personal norms varied based on the source. Normative information from peers had a greater impact on changing personal norms than normative information from teachers. Particularly, while peers and teachers had similar influence in the prosocial domain, adolescents responded differently to risk‐taking norms that signaled greater disapproval than their initial personal norms, depending on the source. Adolescents were more likely to adjust their own risk‐taking norms when the normative information came from peers. This finding aligns with previous research showing that personal norm adjustments are strongest when adolescents observe strong peer disapproval of risk‐taking (Pinho et al., 2021). In contrast, they were more resistant to influence from teachers. A possible explanation for this discrepancy is psychological reactance, the tendency to resist social influence when one's sense of freedom is threatened (Brehm, 1966; Steindl et al., 2015). Adolescents, in particular, may exhibit reactance as they strive for attitudinal freedom and autonomy (Crone & Dahl, 2012; Elder Jr & Shanahan, 2007). Strong disapproval from teachers might be perceived as an imposition, which could explain why interventions from teachers or other adults did not yield strong results (Yeager et al., 2018).

Conversely, in the prosocial domain, adolescents appear to follow both peer and teacher norms. Prosociality is universally valued across all life stages (Chen, 2023; Crone & Achterberg, 2022; Zuffianò et al., 2023), and individuals exhibit such behavior from an early age (Brownell, 2013). Consequently, prosocial normative signals from teachers may carry significant weight, as teachers are part of a broader social network that extends beyond school peers to the larger adult community in which adolescents will soon function. When it comes to widely accepted prosocial behaviors, adolescents may balance peer and teacher norms, aligning with peer groups while also preparing for independent adult roles. These findings suggest that the effects of source on personal norm updates and subsequent intentions may be specific to the behavioral domain.

### Strengths and limitations

Our experimental design allowed us to test the impact of normative information stemming from a group of peers and teachers on personal norm change and intention change in a controlled and rigorous manner. Although participants were exposed to normative information from peers and teachers, their responses were made privately. This likely reduced the influence of the “public” component of social expectations and mere conformity, suggesting that the observed changes cannot be fully attributed to social desirability bias alone. However, while we anonymized the identity of the source (by indicating only a group of peers or teachers), adolescents may have thought of particular teachers or peers, which may have impacted some of the responses. Future empirical work could disclose the identity of the sources and examine their influence, considering that different teachers may impact adolescent responses to normative cues distinctly. For instance, a highly esteemed teacher may be very influential, a factor that could be strategically leveraged in the school context.

Additionally, as a form of control, our study used confirming norm information, where normative information aligned with participants' initial ratings. This approach helped mitigate demand characteristics and ensured that observed changes were not solely due to exposure to distinct normative information. However, we acknowledge that other control conditions, such as a no‐information condition, could help account for naturally occurring norm shifts. Furthermore, while our approach maintained a consistent distance between participants' personal norms and the normative information, real‐world social norms differ in strength and direction. Exploring how variations in the distance and direction of normative information influence behavioral changes, along with incorporating follow‐up measurements to assess the persistence of norm and intention changes over time, could provide a valuable avenue for future research. Although our set‐up may encourage socially responsible behavior depending on the source, real‐life scenarios frequently entail additional factors, such as individuals' confidence (Gradassi et al., 2023; Slagter, Gradassi, et al., 2023; Slagter, van Duijvenvoorde, & van den Bos, 2023), certainty (Reiter et al., 2021; Slagter, Gradassi, et al., 2023; Slagter, van Duijvenvoorde, & van den Bos, 2023), or even time–pressure (Shulman & Cauffman, 2014) which could impact how adolescents use novel information to navigate the social environment. Future research could investigate the impact of these features on intention formation.

### Practical implications

Overall, our findings highlight the important role peers and teachers play in shaping adolescents' own norms and intentions, which is crucial for enhancing a positive school climate and sense of community. Identifying relevant sources of normative information becomes crucial to tailor strategies focused on behavioral changes. For instance, teachers could be equipped with strategies to reinforce prosociality. This could be vital to foster behaviors such as helping, sharing, and cooperating with others not only at the school but also in larger contexts, such as communities. However, interventions aimed at changing adolescents' own norms and subsequent intentions should be cautious about using teachers' risk‐taking norms as this approach may result in resistance or unintended responses. Conversely, peers exert a strong influence, underscoring the importance of leveraging peer dynamics to promote positive change effectively (LaFontana & Cillessen, 2010). A clear understanding of how different normative sources influence adolescents can inform the design of more effective interventions, promoting healthy and socially responsible behaviors during adolescence.

## CONCLUSION

This study investigated how normative information influences adolescents' intention for real‐world behaviors, both directly and indirectly through changes in personal norms. Additionally, we examined how these pathways are shaped by the source of normative influence: peers or teachers. Our findings indicate that normative information directly, and indirectly through updates in personal norms, shapes adolescents' intentions. While both peers and teachers contribute to shaping prosocial intentions, their influence on risk‐taking intentions diverges, with adolescents being more influenced by peers and more resistant to norms communicated by teachers. These results highlight the complex interplay between external normative cues and adolescents' evolving personal norms, underscoring the need to consider both sources of influence when designing interventions to support positive adolescent development.

## AUTHOR CONTRIBUTIONS

**Ana da Silva Pinho:** conceptualization; methodology; investigation; writing – original draft; writing – review and editing; visualization; formal analysis; project administration; data curation; resources; validation; software. **Scarlett Slagter:** writing – review and editing; investigation. **Andrea Gradassi:** writing – review and editing. **Lucas Molleman:** conceptualization; writing – review and editing; methodology; supervision. **Barbara R. Braams:** conceptualization; supervision; writing – review and editing. **Wouter van den Bos:** conceptualization; funding acquisition; writing – review and editing; resources; supervision; methodology.

## FUNDING INFORMATION

European Research Council, Netherlands Organization for Scientific Research.

## CONFLICT OF INTEREST STATEMENT

The authors declare that there are no conflicts of interest associated with this manuscript.

## ETHICS STATEMENT

All procedures were approved by the Ethics Review Board of the Faculty of Social and Behavioral Sciences of the University of Amsterdam (case number 2021‐DP‐14191).

## PATIENT CONSENT STATEMENT

No patients were included in the study.

## Supporting information


Data S1:

## Data Availability

All data and code supporting the findings of this study are available from the public repository, accessible at the Open Science Framework via https://osf.io/4yevs/?view_only=f2884a6fe0f449b18bee217a5c130003.
