# Supplementary material for: Teacher knows best? The social influence of teachers and peers in high school
Source: J Res Adolesc. 2025 Aug 17;35(3):e70063. doi: 10.1111/jora.70063 (PMC12358715; doi:10.1111/jora.70063)
Supplement: Supplementary file 1 — Data S1: [file JORA-35-0-s001.docx]

[**Supplementary figures 3**](#_qt8zohvhhr2o)

[Figure S1.](#_6n8vyhy4qucl)

[Figure S2. 3](#_i3evivplpq8l)

[Figure S3. 4](#_ixf0p79ir1uq)

[Figure S4. 5](#_cje44s402f0p)

[**Supplementary tables 6**](#_v6t1abpe1hcw)

[TABLE S1. Population parameters for power analysis. 6](#_1w2h201cboes)

[TABLE S2. List of behavioural items by domain. 7](#_yxfqy0vqt06u)

[TABLE S3. Impact of normative information separately for risk-taking and prosocial intentions. 8](#_yvoh37ah1bef)

[TABLE S4. Results of the moderating effect of age. 9](#_6atsqhvj0cjf)

[TABLE S5. Results of the moderating effect of subjective closeness. 10](#_ulsz755h91gj)

##

# **Supplementary figures**


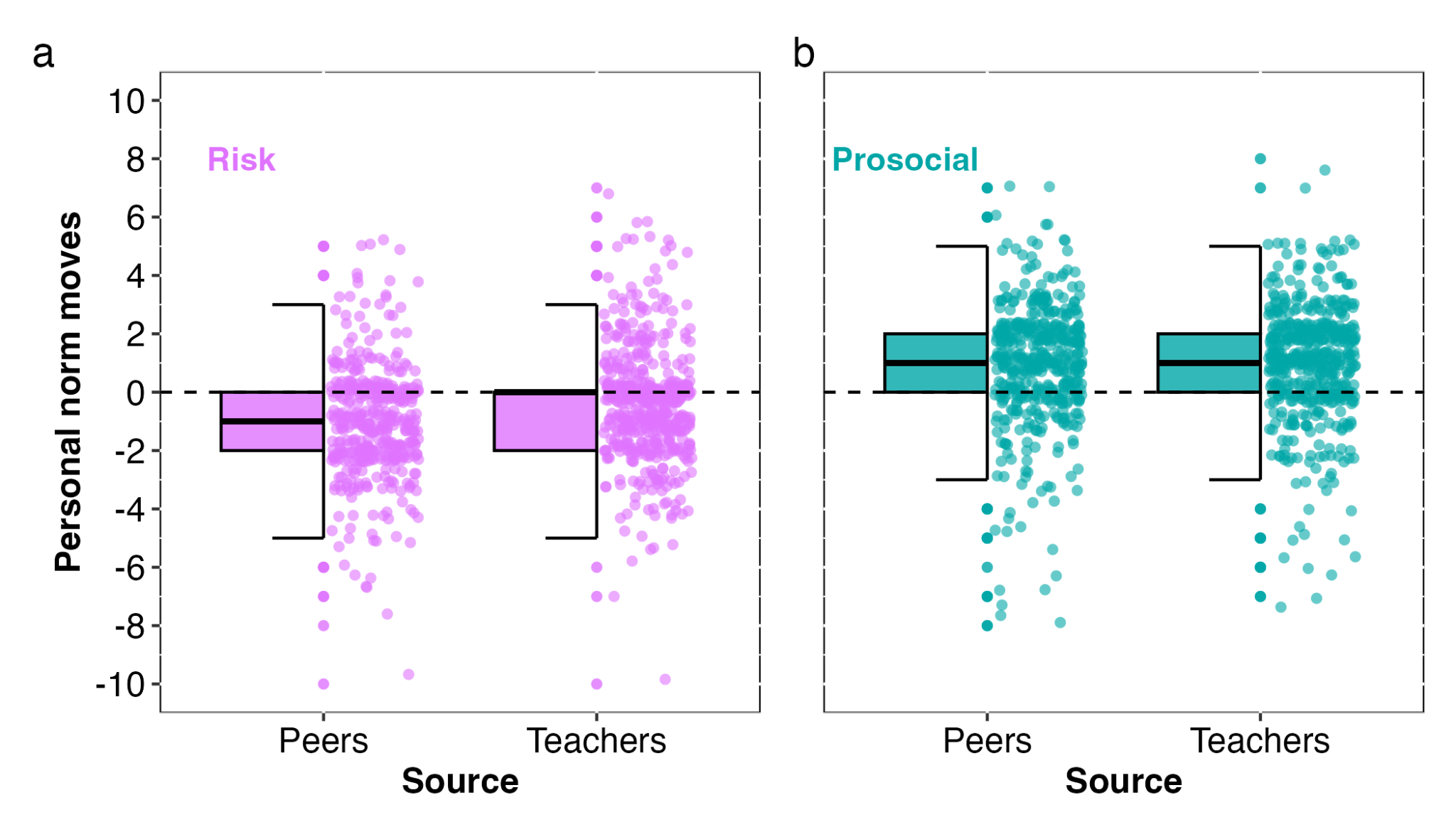


## Figure S1. Frequency distribution of moves in personal moves between wave 1 and 2 across behavioural domain and broken down by source: (a) peers and (b) teachers. The boxplots represent the interquartile range (IQR), the median is represented by the horizontal line and the whiskers include the smallest and largest values within 1.5 times the IQR. Jittered points show individual moves in experimental trials for risk-taking (purple) and prosociality (turquoise).
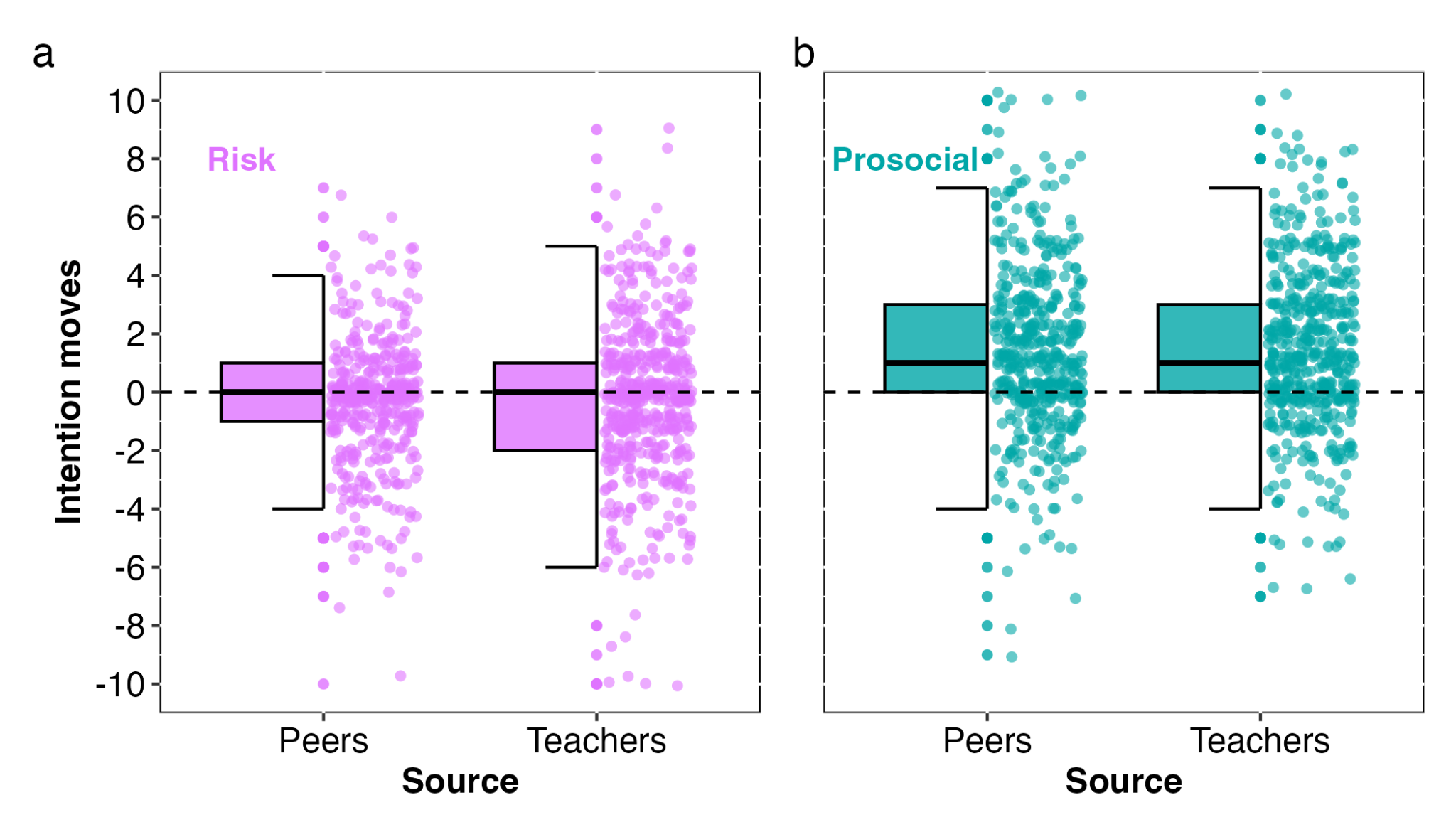


## Figure S2. Frequency distribution of moves in intention between wave 1 and 2 across behavioural domain and broken down by source: (a) peers and (b) teachers. The boxplots follow the same criteria as in Figure S1.

##
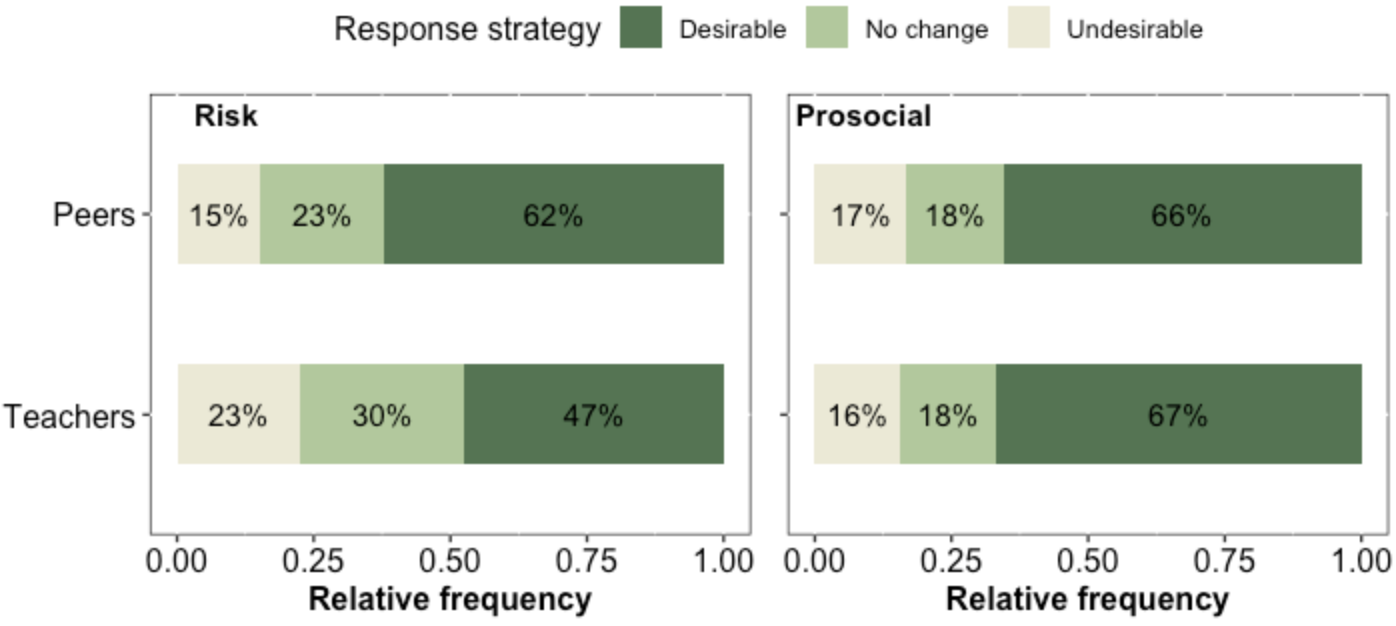


## Figure S3. Response strategies in personal norm updates following exposure to normative information. Responses were categorised into three types: desirable change (shifting towards the observed norm), no change (maintaining one’s initial personal norm), and undesirable change (shifting away from the observed norm, i.e., anticonformity). For risk-taking (left panel), when normative information was provided by peers, participants aligned their personal norms with the observed norm in 62% of trials, maintained their initial norm in 23%, and moved away from the normative information in 15% of cases. When the normative information was provided by teachers, participants adopted the socially desirable norm in 47% of trials, maintained their initial norm in 30%, and moved away in 22% of cases. For prosociality (right panel). For prosociality (right panel), the direction of change in personal norm was comparable between peers and teachers with participants moving toward the socially desirable norm in two-thirds of the cases, keeping it in 18% and moving their initial norm in an undesirable way in 16%.

##


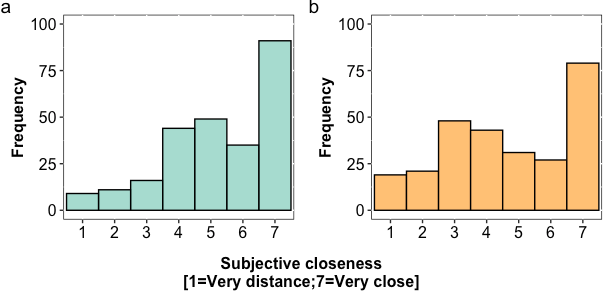


## Figure S4. Frequency distribution of subjective closeness. (a) Closeness to the peer group (*M* = 5.25, *SD* = 1.72) and (b) to the teacher group (*M* = 4.66, *SD* = 1.97). Adolescents reported to feel closer to their peers than to teachers (*U*= 6557.5, *r* = 0.77, *p* < 0.001).

#

# **Supplementary tables**

## TABLE S1. Population parameters for power analysis.

| Parameter | Label | Description | Value |
| --- | --- | --- | --- |
| *ΔIB* ~ *N* | c | ΔIB is regressed on N | 0.3 |
| *ΔIB* ~ *N*source* | cw | ΔIB is regressed on N*source | 0.2 |
| *ΔIB* ~ *ΔPN* | b | ΔIB is regressed on ΔPN | 0.25 |
| ΔPN ~ N | a | *ΔPN* is regressed on N | 0.4 |
| ΔPN ~ N*source | aw | *ΔPN* is regressed on N*source | 0.2 |
| *N*~~N |  | Residual variance of *N* | 4 |
| ΔPN~~ΔPN |  | Residual variance of *ΔPN* | 3 |
| ΔIB~~ΔIB |  | Residual variance of *ΔIB* | 4 |
| N~~*N*source* |  | Residual of N covaries with residual of *N*source* | 1 |
| *N*source*~~*N*source* |  | Residual variance of *N*source* | 1 |
| indirect effect (a*b, when teacher = 0) | ab0 |  | 0.1 |
| indirect effect (ab0+1*aw*b, when peer = 1) | ab1 |  | 0.15 |
| difference between indirect effects (ab1-ab0) | ind.diff |  | 0.05 |
| total effect (ab0+c, when teacher = 0) | total0 |  | 0.4 |
| total effect (ab1+c+1*cw, when teacher = 0) | total1 |  | 0.65 |

*Note. N* = normative information, *ΔIB =* intention change, *ΔPN =* personal norm change.

##

## TABLE S2. List of behavioural items by domain.

| Behavioural domain | | |
| --- | --- | --- |
| Offline  risk-taking | Online  risk-taking | Prosocial |
| Smoking cigarettes (include e-cigarette) | Downloading illegal software or films | Helping others with problems |
| Drinking alcohol | Playing online games | Standing up for a peer who is bullied |
| Getting drunk | Watching pornographic content online | Exchanging meat for veggies 3x a week |
| Smoking weed | Connecting with strangers on online platforms (e.g., Facebook, Instagram) | Striking for climate |
| Truancy | Using parents credit card without permission to buy things online | Recycling |
| Bullying | Sharing one’s opinion online | Donating to charities |
| Cheating on an exam | Watching violent content online | Doing sports |
| Staying up late at night (past 23) during weekdays | Using social media to humiliate a peer | Giving money to homeless |
| Stealing small items from a store | Sharing personal information online (e.g., showing email on a social media platform) | Volunteering work (e.g., scouting) |
| Texting or calling while cycling | Spending more than 4 hours on social media platforms | Sharing things (food, class notes) |
| Snowboarding without protection | Spending all your allowance on online games | Inviting a peer who is not popular/liked to a party/group event |
| Not wearing a helmet on scooter |  | Letting people go ahead of you in a queue |
|  |  | White lie |

## TABLE S3. Impact of normative information separately for risk-taking and prosocial intentions.

|  | **Model 1**  **risk-taking** |  | **Model 2**  **prosociality** |  |
| --- | --- | --- | --- | --- |
|  | **Estimate (*SE*)** | ***p*** | **Estimate (*SE*)** | ***p*** |
| **Direct effect** |  |  |  |  |
| Normative information→ Personal norm change (*a*) | 0.02 (0.37) | .958 | 1.09 (0.30) | < .001 |
| Personal norm change→Intention change (*b*) | 0.39 (0.05) | <.001 | 0.38 (0.05) | < .001 |
| Normative information→Intention change (*c’*) | 0.33 (0.30) | .263 | –0.10 (0.50) | .840 |
| **Indirect effect** |  |  |  |  |
| *a*b* | 0.01 (0.15) | .958 | 0.41 (0.11) | .<.001 |
| **Total effect** |  |  |  |  |
| *c’+ab* | 0.34 (0.34) | .317 | 0.31 (0.52) | .556 |
| **Moderator** |  |  |  |  |
| Source on path c’ | -0.31 (0.18) | .091 | 0.10 (0.34) | .762 |
| **Moderator** |  |  |  |  |
| Source on path *ab* | 0.08 (0.07) | .206 | –0.09 (0.08) | .211 |
| RMSEA | 0 |  | 0 |  |
| CFI | 1 |  | 1 |  |

*Note*. Estimates from the moderated mediation model fitted to changes in risk-taking intentions (Model 1) and prosocial intentions separately (Model 2). Normative information serves as the independent variable, intention change as the dependent variable, and personal norm change as the mediating variable. Source is the moderator. Both models test whether intentions for risk-taking and prosociality are influenced directly by normative information (path c’) and indirectly through personal norm change (mediation), as well as whether these pathways are moderated by the source. Model 1 explained 4.2% of the variance in personal norm change (*R²* = 0.042) and 9.5% of the variance in intentions change (*R²* = 0.095). Model 2 explained 5.9% of the variance in personal norm change (*R²* = 0.059) and 7.4% of the variance in intentions change (*R²* = 0.074). Both models report unstandardized coefficients. *SE* represents standard error.

## TABLE S4. Results of the moderating effect of age.

|  | **Model 1**  **Peers** |  | **Model 2**  **Teachers** |  |
| --- | --- | --- | --- | --- |
|  | **Estimate (*SE*)** | ***p*** | **Estimate (*SE*)** | ***p*** |
| **Direct effect** |  |  |  |  |
| Normative information→ Personal norm change (*a*) | 0.46 (0.03) | <.001 | 0.36 (0.03) | <.001 |
| Personal norm change→Intention change (*b*) | 0.32 (0.04) | <.001 | 0.42 (0.04) | <.001 |
| Normative information→Intention change (*c’*) | 0.24 (0.05) | <.001 | 0.23 (0.04) | <.001 |
| **Indirect effect** |  |  |  |  |
| *a*b* | 0.14 (0.02) | <.001 | 0.15 (.02) | <.001 |
| **Total effect** |  |  |  |  |
| *c’+ab* | 0.38 (0.04) | <.001 | 0.38 (0.04) | <.001 |
| **Moderator** |  |  |  |  |
| Age on path c’ | -0.02 (0.11) | .837 | -0.09 (0.03) | .002 |
| **Moderator** |  |  |  |  |
| Age on path *ab* | -0.00 (0.01) | .874 | –0.02 (0.01) | .174 |
| RMSEA | 0 |  | 0 |  |
| CFI | 1 |  | 1 |  |

*Note.* Model 1 tests whether age moderates the direct and indirect paths from normative information from peer to intentions. Model 2 tests whether age moderates the direct and indirect paths from normative information from teachers to intentions. Both models report unstandardized coefficients. *SE* represents standard error.

## TABLE S5. Results of the moderating effect of subjective closeness.

|  | **Model 1**  **Peers** |  | **Model 2**  **Teachers** |  |
| --- | --- | --- | --- | --- |
|  | **Estimate (*SE*)** | ***p*** | **Estimate (*SE*)** | ***p*** |
| **Direct effect** |  |  |  |  |
| Normative information→ Personal norm change (*a*) | 0.46 (0.04) | <.001 | 0.36 (0.03) | <.001 |
| Personal norm change→Intention change (*b*) | 0.33 (0.04) | <.001 | 0.43 (0.05) | <.001 |
| Normative information→Intention change (*c’*) | 0.22 (0.04) | <.001 | 0.24 (0.05) | <.001 |
| **Indirect effect** |  |  |  |  |
| *a*b* | 0.15 (0.02) | <.001 | 0.15 (0.02) | <.001 |
| **Total effect** |  |  |  |  |
| *c’+ab* | 0.37 (0.04) | <.001 | 0.39 (0.05) | <.001 |
| **Moderator** |  |  |  |  |
| Subjective closeness on path c’ | 0.02 (0.01) | .705 | 0.05 (0.03) | .044 |
| **Moderator** |  |  |  |  |
| Subjective closeness on path *ab* | 0.00 (0.01) | .879 | -0.02 (0.01) | .111 |
| RMSEA | 0 |  | 0 |  |
| CFI | 1 |  | 1 |  |

*Note.* Model 1 tests whether perceived closeness to the source from which the norm was derived moderates the direct and indirect paths from normative information from peers to intentions. Model 2 tests whether perceived closeness moderates the direct and indirect paths from normative information from teachers to intentions. Both models report unstandardized coefficients. *SE* represents standard error.
